# Physical therapy using digital therapeutics in infants with congenital muscular torticollis: a pilot study

**DOI:** 10.1038/s41598-026-56419-3

**Published:** 2026-06-14

**Authors:** Chang Hee Lee, Jecheon Seong, Yun Jee Lee, Dasom Park, Aram Kim

**Affiliations:** 1 Department of Rehabilitation Medicine, Myongji Hospital, 14-55 Hwasu-Ro, Deokyang-Gu, Goyang-Si, Gyeonggi-Do 412-270 Republic of Korea; 2 Department of Rehabilitation Medicine, Myongji Hospital, Hanyang University College of Medicine, 14-55 Hwasu-Ro, Deokyang-Gu, Goyang-Si, Gyeonggi-Do 412-270 Republic of Korea

**Keywords:** Congenital muscular torticollis, Physical therapy, Digital therapeutics, Artificial intelligence, Paediatric research, Rehabilitation, Biomedical engineering

## Abstract

Congenital muscular torticollis (CMT) is a common musculoskeletal problem associated with unilateral sternocleidomastoid muscle stiffness. We evaluated the feasibility of the newly developed digital therapeutics (DTx) ACESO-APP, a personalized exercise app with real-time feedback, using skeleton-based motion analysis and artificial intelligence-driven body reconstruction models. Fourteen participants were randomized to undergo either weekly PT with standard home program education (SE group) or weekly PT with digital therapeutics lead home program (DTx group). A 12-week home exercise program (HEP) comprising stretching exercises was encouraged in both groups with weekly in-hospital PT. Clinical changes were assessed using the torticollis overall assessment at 4, 8, and 12 weeks, and participants’ HEP adherence over 1 week was assessed using a questionnaire. Recruitment and completion rates, adherence rate, System Usability Scale scores, and adverse events were summarized using descriptive statistics. Eight participants (4 in each group) completed the study. The overall recruitment rate was approximately 5.2 participants per month, and the completion rate was 57.1% for both groups. The DTx group demonstrated higher adherence, with no reported adverse events. ACESO-APP demonstrated feasibility but achieved a suboptimal completion rate. These preliminary findings support large-scale trials and further optimization of HEP using the ACESO-APP in children with CMT.

## Introduction

Congenital muscular torticollis (CMT) is a postural and musculoskeletal deformity that occurs after birth and is the third most common congenital musculoskeletal problem in newborns^1^. Patients with CMT have unilateral stiffness of the sternocleidomastoid (SCM) muscle, resulting in lateral flexion of the head to the ipsilateral side and rotation to the contralateral side, and commonly exhibit a restricted range of cervical motion^1,2^. Substantial evidence indicates that early intervention with physical therapy (PT) greatly improves symptoms; however, delayed intervention is associated with a prolonged treatment duration^3,4^. The primary intervention for CMT involves stretching exercises targeting the affected SCM, with evidence indicating that the total amount of exercise is a key factor in symptom alleviation^5^.

As the amount of PT available in hospitals or specialized treatment centers is often limited, a structured home exercise program (HEP) for CMT may increase the total amount of intervention a child receives^6^. Parents often seek medical information on the treatment of their children with CMT on the Internet. A previous study evaluated the usefulness, reliability, and quality of Internet-based video clips on CMT and reported that video clips presented by healthcare professionals were more useful, reliable, and of higher quality than popular videos by non-healthcare professionals^7^. These findings highlight the need for accessible and high-quality treatment guidance provided by healthcare professionals. Therefore, in this study, we applied the concept of digital therapeutics, whereby physicians prescribed an HEP through an application for the appropriate management of CMT.

Digital therapeutics (DTx) can be defined as “therapeutic interventions through a clinically evaluated, patient-directed-software application intended to improve the process of disease diagnosis, treatment, management, and prevention”^8^. The development of therapies incorporating the concept of DTx has increased in recent years, particularly those targeting chronic and behavior-modifiable conditions^8^. This offers the potential for improved cost-effectiveness by facilitating treatment outside healthcare facilities and allowing patients to use computers, smartphones, or tablets for therapy in any setting. A few studies have investigated the application of DTx in children with behavioral problems, such as autism spectrum disorder (ASD) and attention deficit hyperactivity disorder (ADHD), with Food and Drug Administration authorization currently available for pediatric ADHD. Digital interventions can facilitate ASD diagnosis in primary care settings and may have beneficial effects on attention, working memory, and inhibitory control in children with ADHD^9–11^. To the best of our knowledge, no previous study has investigated the use of DTx in the treatment of CMT. Since most infants with CMT have a favorable prognosis after early, appropriate, and highly intensive PT^12^, evidence-based DTx prescribed by physicians may be an appropriate option for improving adherence to exercise. Our newly developed multiplatform app “ACESO” DTx (ACESO-APP) utilized advanced artificial intelligence (AI) such as skeleton-based motion analysis and AI-driven body reconstruction models. Part of the DTx uses AI to provide customized and adaptive algorithms and interventions, for example, in a musculoskeletal disease entity. Kaia Health developed an AI-based computer vision technology that enables smartphones and tablets to analyze body movement patterns during exercises and provides corrective feedback for back pain^8,13,14^. By implementing AI-based DTx in infants with CMT, we were able to measure and analyze movement data, such as cervical range of motion and provide real-time corrective feedback without wearable devices.

Accordingly, this study was designed as a pilot feasibility trial with the primary aim of evaluating the recruitment rate, completion rate, adherence rate, usability, and safety of an HEP delivered via the ACESO-APP. In addition, we collected preliminary data on clinical outcomes as measured using the Torticollis Overall Assessment (TOA)^15^ over 12 weeks (T0, baseline; T1, 4 weeks; T2, 8 weeks; and T3, 12 weeks) to inform the design of future large-scale studies.

## Results

### Patient characteristics

Fourteen participants were screened, enrolled, and randomized into the DTx (n = 7) and SE (n = 7) groups. Eight participants (n = 4 in SE and n = 4 in DTx) completed the intervention and assessment and consistently attended the in-hospital PT sessions. The demographic data of the participants are presented in Table 1. The baseline characteristics were generally comparable between the two groups.

**Table 1:** Baseline characteristics of participants (n = 14).

		**DTx group (n = 7)**	**SE group (n = 7)**
Age (days)^a^		160.4 ± 94.0	146.5 ± 54.2
Sex^b^	Female	4(57)	4(57)
	Male	3(43)	3(43)
Involved side (left)^b^	Left	5(71)	5(71)
	Right	2(29)	2(29)
TOA score (points)^a^	Total	12.8 ± 2.6	12.7 ± 2.9
	Rotation deficits	2.8 ± 0.3	3.0 ± 0.0
	Side flexion deficits	2.5 ± 0.5	2.4 ± 0.5
	Craniofacial asymmetry	2.2 ± 0.7	1.8 ± 0.3
	Residual band	3.0 ± 0.0	2.2 ± 1.2
	Head tilt	1.4 ± 0.7	1.4 ± 0.7
	Subjective assessment by parents	1.7 ± 1.2	1.7 ± 1.2

^a^Mean ± standard deviation. ^b^n (%).

### Feasibility of ACESO-APP

#### Recruitment and completion/attrition rate

**Fig. 1:**
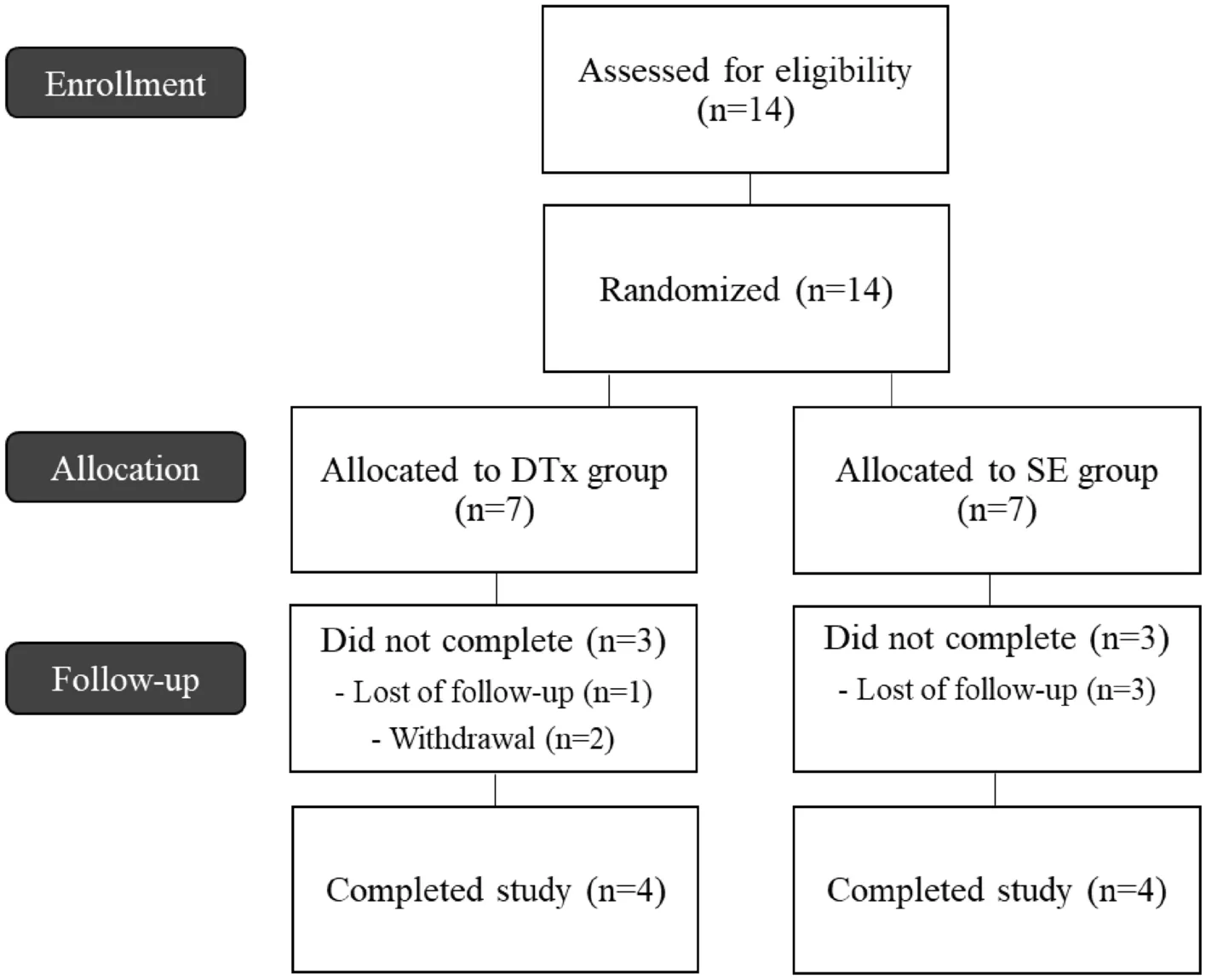
CONSORT flow diagram of participant flow in the feasibility study.

Between December 12, 2023 and March 4, 2024, 14 participants were screened for eligibility, with seven participants in each group. The recruitment rate during the study period was approximately 5.2 participants per month. In the DTx group, four of seven participants completed the study (completion rate: 57.1%), with an attrition rate of 42.9% (3/7). The completion rate was the same as that in the SE group (57.1%). Six participants discontinued the study. In the DTx group, three participants withdrew (loss to follow-up, n = 1; poor medical condition of the infant, n = 1; technical difficulty, n = 1). In the SE group, three participants were lost to follow-up. The participant flowchart is presented in Fig. 1.

#### Adherence rate

The adherence rate was 42.9% (3/7) in the DTx group and 28.6% (2/7) in the SE group, defined as the proportion of participants achieving at least 80% of the prescribed frequency of seven sessions per week, based on self-reported data collected via a weekly questionnaire. Exercise amount among participants who completed the study is presented in Table 2.

**Table 2:** Adherence to the home-based exercise program (exercise amount) among participants who completed the study (n = 8).

	DTx group (n = 4)			SE group (n = 4)		
	Frequency (session/week)	Duration (min/session)	Total dosage (min/week)	Frequency (session/week)	Duration (min/session)	Total dosage (min/week)
4 weeks (T1)	10.2 ± 3.3	38.1 ± 27.6	368.7 ± 352.0	7.5 ± 3.3	14.8 ± 13.5	128.3 ± 130.9
8 weeks (T2)	7.7 ± 4.5	20.0 ± 26.7	143.7 ± 185.9	10.2 ± 9.0	18.7 ± 13.1	106.2 ± 54.3
12 weeks (T3)	8.6 ± 6.2	19.0 ± 21.1	167.3 ± 164.6	8.0 ± 9.0	12.5 ± 11.9	60.0 ± 44.9

#### Usability and satisfaction

**Fig. 2:**
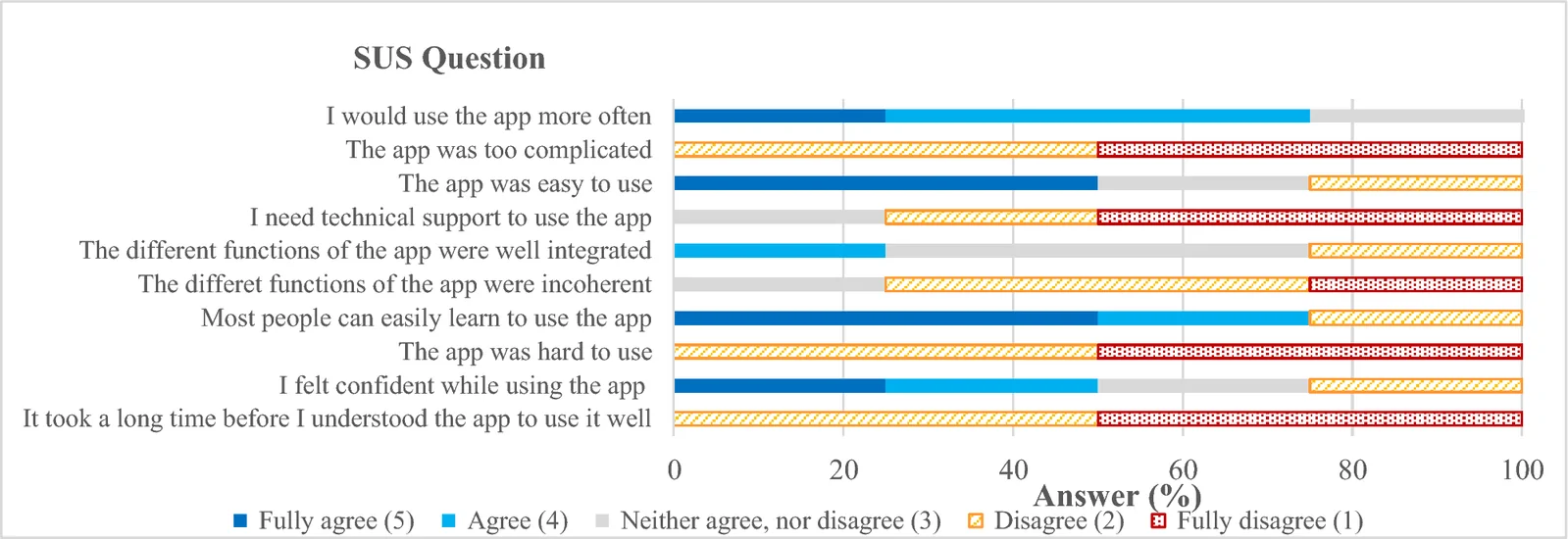
Scores in the SUS of ACESO-APP.

Regarding usability, the mean System Usability Scale (SUS) score^16^-a widely used self-administered instrument for assessing system usability-was 73.1 (Grade “B”), indicating good usability; The SUS scores range from 0 to 100, with higher scores reflecting better usability (Fig. 2). Participant satisfaction was measured using a 7-point Likert scale. The highest score was observed for “interest” (5.2 ± 1.2), followed by “stability” (5.0 ± 2.1), “satisfaction” (5.0 ± 1.4), “role expectation for rehabilitation” (5.0 ± 1.4), “symptom improvement” (4.7 ± 0.9), “intention to use” (4.7 ± 1.7), “adequate difficulty” (4.2 ± 2.0), and “physical comfort” (3.5 ± 1.2).

#### Safety

No adverse symptoms were reported in either group.

#### Exploratory clinical outcomes

**Fig. 3:**
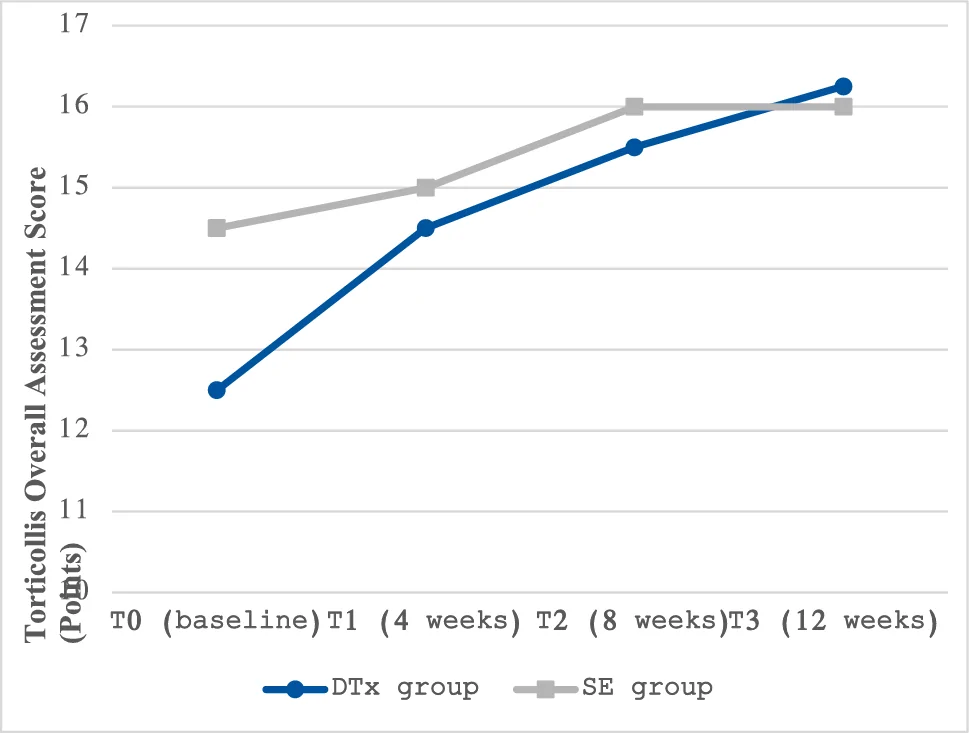
Torticollis overall assessment score in the DTx and SE groups among participants who completed the study.

Exploratory analyses were performed to assess clinical outcomes (TOA scores) in DTx and SE groups among participants who completed the study. In the DTx group, the mean of TOA scores improved from 12.0 ± 3.3 at baseline to 16.2 ± 2.3 at the end of the study, whereas the SE group showed a change from 14.5 ± 2.3 to 16.0 ± 2.7 (Table 3 and Fig. 3). However, these changes were not statistically tested because of the small sample size.

**Table 3:** Changes in clinical outcomes: total torticollis overall assessment scores in the DTx and SE groups among participants who completed the study (n = 8).

Time point	DTx group (n = 4, points)	SE group (n = 4, points)
Baseline (T0)	12.5 ± 3.3	14.5 ± 2.3
4 weeks (T1)	14.5 ± 2.0	15.0 ± 2.1
8 weeks (T2)	15.5 ± 1.7	16.0 ± 1.4
12 weeks (T3)	16.2 ± 2.3	16.0 ± 2.7

Mean ± standard deviation. DTx, digital therapeutics; SE, standard education.

## Discussion

Research on DTx-utilizing wearable devices has advanced considerably in recent years, and numerous sensors have been employed to build wearable systems for human movement monitoring. These sensors recognize human motion data, analyze it, and simulate correct postures. These systems often rely on inertial and electromyography (EMG) sensors to detect body movements^17,18^. However, in the case of DTx using wearable devices, some limitations in terms of wearable comfort and security (e.g., wearable electronic products), especially in infants were noted^17^. This study introduces a noninvasive DTx that uses skeleton-based motion analysis and AI-driven body reconstruction models. To analyze body movements without wearable devices, cameras or vision sensors must be used to track the skeleton. A skeleton-based motion analysis model converts 2D skeleton data into 3D skeleton data^14,17^. An AI-based body reconstruction model uses AI techniques such as deep learning to reconstruct or analyze a user’s body^19^. This AI model technique enables continuous AI feedback, assisting users to improve their posture and movement throughout the PT process. For example, previous studies have developed customized programs using AI-based computer vision technology, which analyze movement patterns during PT and provide corrective feedback on back pain^13,14^. Even though quantitative measurement such as marker-based 3D motion analysis is considered the gold standard for motion tracking, it is time-consuming for accurate placement of markers and requires patients’ cooperation^20,21^. These limitations can be overcome using a red–green–blue depth (RGBD) camera with integrated 3D body-tracking functionality. These cameras are portable, affordable, and do not require expensive facilities or specialized personnel. The previous studies introduced a deep-learning model converting 2D skeleton data from RGBD cameras to 3D models to monitor patient movement for telerehabilitation with high accuracy^22,23^. Another study has reported that this system was valid and accurate for various movement tasks in children^24^. In this study, to improve the accuracy of inferring 3D movements, an AI model was trained using images captured by RGB-D cameras. However, our system requires AI to differentiate between the child and the caregiver during the interaction. This additional requirement introduces greater complexity than the approaches that focus solely on the movement analysis of a single individual. Despite this challenge, our approach enabled differentiation between the child and caregiver, and analyzed only the child’s movements by applying an AI model trained using 300,000 pieces of physical and behavioral data of children in daycare centers, and an algorithm developed and added to the existing model. The AI model may provide a convenient, safe, personalized, and real-time feedback system for corrective exercises, ultimately enhancing the therapeutic effectiveness. It can also be cost-effective and increase healthcare accessibility for patients who experience difficulties in contacting medical services. DTx without wearable devices may be a promising treatment strategy for children, particularly infants.

This study aimed to evaluate the feasibility of ACESO-APP, which is the DTx providing home-based, AI-driven, and not-wearable device HEP with real-time feedback for corrective motion in infants with CMT. This pilot study demonstrated that the ACESO-APP has acceptable feasibility, as reflected by high recruitment, higher adherence in the DTx group, favorable usability with the exception of a suboptimal completion rate, and absence of adverse events. Clinical outcomes improved in both the DTx and SE groups, as evidenced by increases in TOA scores among the participants who completed the study. Although these changes were not statistically tested owing to the small sample size, they suggest potential trends that warrant further investigation in future studies.

The primary intervention in infants with CMT involves stretching exercises targeting the affected SCM. Stretching exercises can be performed with the infant in a supine or seated position depending on the caregiver’s lap^25^. In this study, the exercises were guided by an application requiring a front-facing camera setup for motion tracking. Considering the potential risks associated with camera placement in the supine position, this study was designed to facilitate exercises in the seated position. Most importantly, the key factors determining the prognosis in CMT treatment are the intensity of the intervention and adherence to the HEP^1,5^. Because the dosage of PT available in hospitals is often limited, structured HEP may increase the overall amount of exercise^6^. Therefore, ACESO-APP is a suitable treatment option for infants with CMT. Based on the satisfaction survey results, the interesting properties of the ACESO-APP may have contributed to adherence to exercise for 12 weeks.

This study has some limitations. First, the study had a small sample size and high dropout rate. Therefore, formal statistical analyses were not feasible, and only exploratory analyses were performed. The high dropout rate likely reflects the inclusion of very young children (including neonates) and the mild nature of the disease, which often leads to the early discontinuation of treatment as symptoms improve. One dropout was attributable to a technical login error, highlighting a limitation of the ACESO-APP in terms of its technical feasibility. Therefore, future studies with larger sample sizes that account for anticipated dropouts and in more stable technical environments with improved system quality should be conducted. Second, although the adherence rate was higher in the DTx group than in the SE group, the adherence rate over the initial 4 weeks showed a declining trend at weeks 8 and 12. The decline in exercise amount suggests that sustaining long-term engagement was challenging despite the high initial participation driven by the novelty of the application. This may be related to factors, such as declining motivation, real-world feasibility, or the repetitive nature of the program. Although a visual summary of the exercises performed that day (number of sets) was provided to support motivation, this measure alone appeared insufficient. Accordingly, alarm-based push notifications and visual feedback for symptom improvement are considered to enhance motivation. The app required caregivers to position the infants and perform exercises in front of the camera, occasionally necessitating a second caregiver. These practical demands may limit the real-world feasibility and sustained adherence. Future research should investigate approaches to reduce caregiver burden and motivate support. In addition, in the advanced version of DTx, a more extensive list of exercises (stretching and strengthening exercises) should be incorporated, encompassing a wider range of scenarios, such as exercises with infants in the supine position. Third, this study was based on the previously validated motion parameters of an RGB-D camera-based markerless 3D motion tracking method reported in previous studies^23,24^^.^ Therefore, a cross-sectional study to directly evaluate the validity of the ACESO-APP and its ability to accurately classify correct and incorrect exercise performance (i.e., accuracy, sensitivity, and specificity) was not conducted, which should be considered a limitation of this study. Fourth, although the TOA serves as a composite instrument reflecting the overall clinical status and is frequently used as a total outcome measure in CMT studies^26,27^, its validity has not been fully established, representing a limitation of this study. Finally, HEP adherence was assessed using a self-administered questionnaire in both groups, which was inherently subjective. Future studies should consider using app-based login records to objectively track adherence.

We developed a DTx ACESO-APP (individualized, home-based, AI-driven, and real-time feedback) and preliminarily evaluated its feasibility and exploratory clinical outcomes as an additional or alternative treatment option for infants with CMT. We will continue to optimize the newly developed ACESO-APP and evaluate its efficacy in large-sample randomized controlled studies.

## Materials and methods

### Study procedures

This prospective, open-label, randomized controlled trial was performed at a single center. Participants were randomly assigned in a 1:1 ratio to either the HEP using the ACESO-APP (DTx group) or standard education (SE group). Block randomization was conducted by an independent staff member using a computer program, and participant enrollment was performed by coordinators who were not involved in sequence generation, preventing investigators from anticipating group assignments. Owing to the distinct nature of the interventions, blinding of participants and providers was not feasible, and the study was conducted as an open-label trial.

### Participants

Participants were recruited from an outpatient pediatric rehabilitation clinic. The inclusion criteria were as follows: (1) CMT diagnosis, (2) age < 12 months, and (3) caregivers who could read, write, and operate general devices (e.g., smartphones or tablets) to use the application. The exclusion criteria were as follows: (1) congenital anomalies of the cervical spine, (2) spasmodic torticollis, (3) neurogenic torticollis, and (4) use of any other therapy for torticollis. An adequate sample size was defined as 14 considering a 20% dropout rate in the pilot study. This study was approved by the institutional review board of our hospital (MJH 2023–07-007, 26/07/2023), and written informed consent was obtained from the parents or legal guardians of all participants. The trial was registered with the Clinical Research Information Service (CRIS; KCT0011438, 09/01/2026).

### Overall description of ACESO-APP

ACESO is a multi-platform application for iOS and Android. Clinical use is available through the Google Play Store and the ACESO App is free to download. The ACESO Web is provided to a physician who prescribes the details of the exercise, including the direction of exercise (according to the affected side), treatment duration (prescription days), and list of exercise types (tilt and rotation), and then permits participants to use the ACESO-APP. ACESO-APP includes three categories: (1) daily measurements, (2) exercise, and (3) statistical data. The degree of cervical tilt was measured daily before the exercise using app. During the exercise, the number of repetitions and real-time feedback for corrective movements was recorded. The amount of daily exercise was recorded and displayed to participants.

### Technology of ACESO-APP

The AI model of ACESO comprises skeleton-based motion analysis and AI-driven body reconstruction models. The motion data from the camera were transmitted to the AI server during daily measurements and exercise and were converted into 3D motion data using the AI model of ACESO. Existing AI models recognize the skeletons and joints of adults; however, to apply the AI model to infants, it was additionally trained using 300,000 pieces of physical and behavioral data of infants and toddlers (0–3 years old) in daycare centers. An algorithm was developed and added to the existing model to advance the technique for extracting data from only a single participant (baby) among the two participants (baby and caregiver) in the video was applied. The AI model was developed based on the OpenPose framework^28^, with adults and pediatric participants annotated separately to enable the model to learn distinct representations for each group. The system detects up to two heads, two shoulder widths, and four hands per frame, and uses skeletal keypoints to calculate arm length and facial proportions to distinguish caregivers from pediatric participants during exercise. To recognize correct and incorrect exercise performance through accurate 3D motion inference, the AI model was trained using 100,000 images of stretching exercises captured with an RGBD camera. The 2D skeleton data of the infants captured by the monocular camera were inferred through a reinforcement learning process on both video sets annotating the infant’s motion and the pseudo-labeled video sets. The inferred 2D skeletons were sent to the server, and the captured video was deleted to protect user privacy. The 2D skeleton data sent to the server were converted into 3D skeleton data through a temporal–spatial attention-GCNFormer network combining motionBERT^29^ and motion AGFormer^30^ using an RGBD camera^24^.

### Intervention

All participants were randomly allocated to one of two groups for 12 weeks: SE or DTx. Weekly manual stretching (PT) was performed by an experienced physiotherapist in both groups. Additional HEP were provided with standard education in the SE group and ACESO-APP in the DTx group. The HEP protocol comprised a lateral tilt stretching exercise on the unaffected side and a rotation stretching exercise on the affected side. Based on the treatment guidelines for CMT, the stretching exercise protocol consisted of 10 repetitions with a 10-s hold for each exercise in both groups, and seven sessions per week were recommended^1,25^.

#### SE group

The caregivers in the SE group were educated on how to perform the HEP and received an exercise instruction brochure. At each follow-up visit, the caregivers were monitored to ensure that the HEP was performed correctly and in accordance with the educated protocol.

#### DTx group

**Fig. 4:**
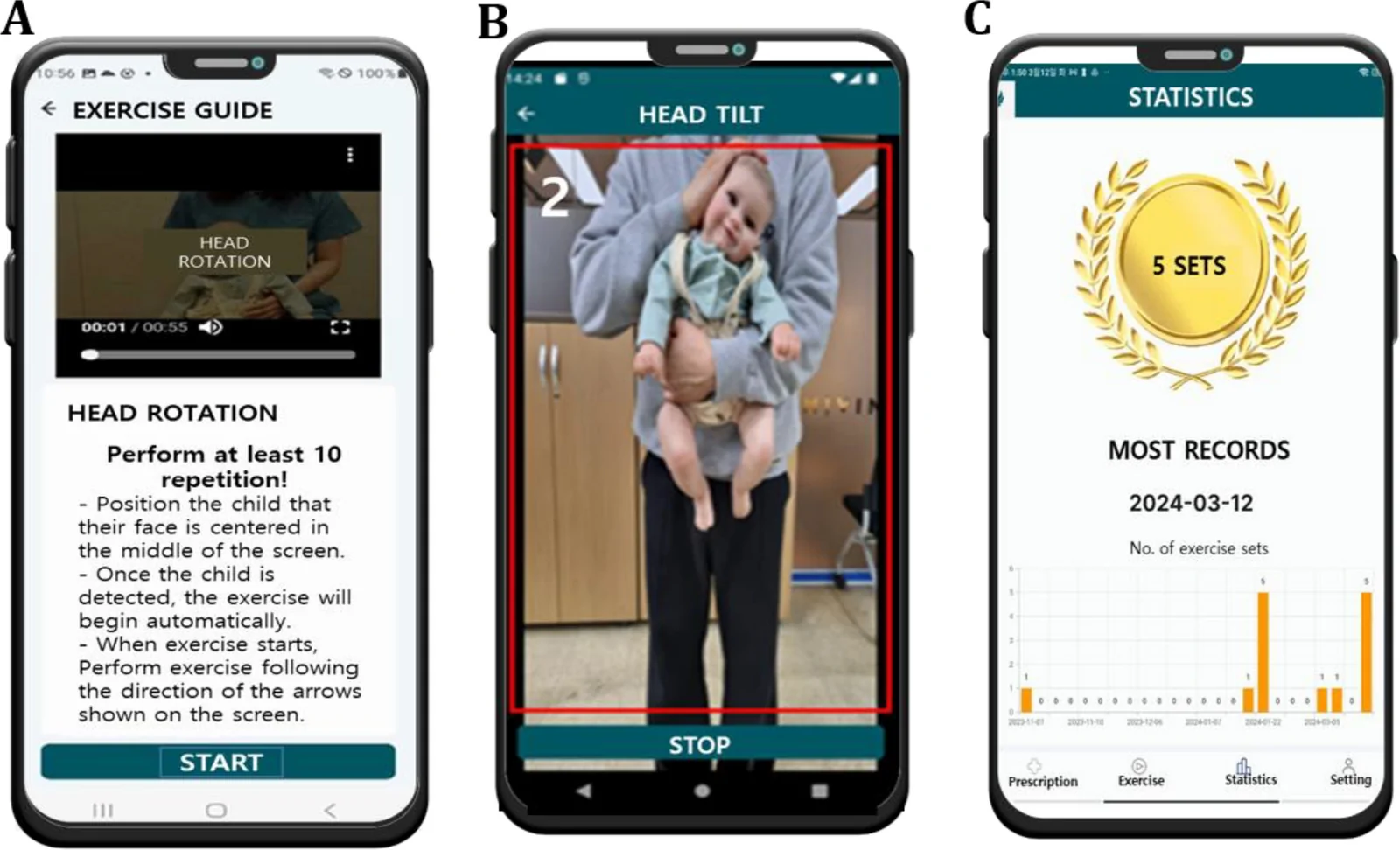
Screenshots of ACESO-APP: (**A**) exercise guide screen, (**B**) exercise screen, (**C**) Statistic data screen (a record of the number of sets). The infant shown in the figure is a mannequin (doll).

The DTx group received a tablet with the ACESO-APP installed and participated in the HEP prescribed by a physician using the app. The physician prescribed the HEP, including the direction of exercise, treatment duration, and list of exercise types, to each participant and then obtained permission to use the ACESO-APP. Before starting the exercise, the degree of cervical tilt was measured daily while keeping their gaze focused. The application shows a list prescribed by a physician and provides an exercise guide video before starting HEP. The number of repetitions was counted during the exercise, and the application notified the participant of errors in the exercise performance using a real-time AI server that converted the camera’s 2D movement data into 3D movement data. Visual summary of the exercise performed that day to help participants monitor their progress and motivate their participation in the HEP throughout the treatment process (Fig. 4).

### Outcome measure

Clinical improvement and amount of exercise (using a self-report questionnaire) were assessed at baseline and at 4, 8, and 12 weeks after the treatment program. The presence or absence of adverse events was assessed at each visit. Furthermore, usability and satisfaction with the ACESO-APP were evaluated using a self-completed questionnaire at the final visit in the DTx group.

#### Clinical assessment

We used the Cheng method^15^, which represents the severity of CMT using the TOA score. It compromises the following details: deficit in cervical rotation (degrees), deficit in cervical lateral flexion (degrees), craniofacial asymmetry, location of the residual band, and subjective assessment by a parent. The detailed results were scored on a 4-point scale: excellent (3), good (2), fair (1), and poor (0). The overall scores were grouped as excellent (16–18), good (12–15), fair (6–11), or poor (< 6), with higher scores indicating better clinical results. Deficits in rotation and lateral flexion were measured using an arthrodial protractor, with the child in the supine position. Limitations in the passive ranges of cervical and lateral flexion were assessed and compared with those on the normal side.

#### Total dosage of exercise

The total dose of HEP per week was evaluated using a self-administered questionnaire. The amount of exercise was estimated in terms of frequency (sessions per week) and duration (minutes per session), and the total dosage of exercise (minutes per week) was calculated by multiplying the frequency and duration.

#### Usability and satisfaction of HEP using ACESO-APP

The Usability of ACESO-APP was evaluated using the standard SUS. The SUS is a widely used self-administered instrument to assess the usability of a system or product through a series of standardized questions on a five-point Likert-type scale. The SUS scores ranged from 0 to 100, with higher scores indicating better results^16,31^. Satisfaction with HEP using the ACESO-APP was evaluated using a study-specific self-report questionnaire that included the following eight items: symptom improvement, interest, physical comfort, stability, satisfaction, intention to use, and role expectations for rehabilitation, with a 7-point Likert-type scale and higher scores indicating better results. The authors developed a 7-point Likert-type scale to assess participant satisfaction. The scale ranged from 1 (“strongly disagree”) to 7 (“strongly agree”), with higher scores indicating greater agreement. Rehabilitation experts reviewed the items to ensure content validity.

#### Safety

Safety was evaluated at each outpatient visit based on the caregiver and physician reports. Adverse events were monitored and documented during the study period.

### Statistical analysis

Feasibility outcomes (recruitment, completion, and adherence rates) were analyzed according to the intention-to-treat principle, including all enrolled participants in their originally assigned groups. Participants with missing data were retained in the denominator, and no imputation was performed. Clinical outcomes were analyzed using a per-protocol approach, which included only participants who completed the study in accordance with a predefined protocol. All statistical analyses were performed using SPSS Statistics version 25 for Windows (IBM Corp, Armonk, NY, USA). The baseline characteristics, clinical improvement, and amount of exercise in the SE and DTx groups were compared. The recruitment and completion rates, adherence rates, and SUS scores were summarized using descriptive statistics, including proportions or means with standard deviations, as appropriate. Considering the pilot nature of the study and its limited sample size, formal hypothesis testing was not performed.

## Data Availability

Data will be available on request, by contacting the corresponding author.

## Abbreviations

CMT: Congenital muscular torticollis
SCM: Sternocleidomastoid
PT: Physical therapy
HEP: Home exercise program
DTx: Digital therapeutics
ASD: Autism spectrum disorder
ADHD: Attention deficit hyperactivity disorder
SE: Standard education
TOA: Torticollis overall assessment
AI: Artificial intelligence
SUS: System usability scale
EMG: Electromyography
RGBD: Red green blue depth
